# Role of Gut Microbiota in Rheumatoid Arthritis

**DOI:** 10.3390/jcm6060060

**Published:** 2017-06-09

**Authors:** Yuichi Maeda, Kiyoshi Takeda

**Affiliations:** 1 Laboratory of Immune Regulation, Department of Microbiology and Immunology, Graduate School of Medicine, WPI Immunology Frontier Research Center, Osaka University, Suita 565-0871, Japan; ymaeda@imed3.med.osaka-u.ac.jp; 2 Core Research for Evolutional Science and Technology, Japan Agency for Medical Research and Development, Tokyo 100-0004, Japan; 3 Department of Respiratory Medicine and Clinical Immunology, Osaka University Graduate School of Medicine, WPI Immunology Frontier Research Center, Osaka University, Suita 565-0871, Japan

**Keywords:** rheumatoid arthritis, microbiota, *Prevotella copri*, gut, Th17 cell

## Abstract

Rheumatoid arthritis (RA) is a systemic autoimmune disease, caused by both genetic and environmental factors. Recently, investigators have focused on the gut microbiota, which is thought to be an environmental agent affecting the development of RA. Here we review the evidence from animal and human studies that supports the role of the gut microbiota in RA. We and others have demonstrated that the abundance of *Prevotella copri* is increased in some early RA. We have also used gnotobiotic experiments to show that dysbiosis in RA patients contributed to the development of Th17 cell-dependent arthritis in intestinal microbiota-humanized SKG mice. On the other hand, *Prevotella histicola* from human gut microbiota suppressed the development of arthritis. In summary, *Prevotella* species are involved in the pathogenesis of arthritis.

## 1. Introduction

The human intestine contains over 1000 bacterial species and 10^14^ bacterial cells [1], but it is difficult to detect most anaerobic bacteria with culture techniques. However, the new sequencing technology, known as “high-throughput microbial DNA sequencing”, has allowed us to better characterize the human microbiota. Recent advances in our understanding of mucosal immunity have clarified the correlation between the gut microbiota and the host immune system [2,3]. Most of the interactions between a host and its commensal microbiota are symbiotic. However, microbial abnormalities, called “dysbiosis” are thought to correlate with various kinds of diseases. Unsurprisingly, intestinal dysbiosis was observed in inflammatory bowel disease [4,5]. Interestingly, dysbiosis is also found in diseases that affect the tissues out of the gut. Recent studies have suggested that the composition of the microbiota is altered in type 1 diabetes, multiple sclerosis, and autism [6,7,8]. Moreover, dysbiosis has been reported in patients with rheumatoid arthritis (RA) in the United States, China, and Finland [9,10,11]. 

Here, we review previous findings regarding the role of microbiota in human RA and mouse models of arthritis. We have shown that some Japanese RA patients carry increased numbers of *Prevotella copri* in the intestine and have used gnotobiotic tools to show that a *P. copri*-dominated microbiota induced Th17 cell-dependent arthritis in mice [12]. In contrast, another study showed that *P. histicola* suppresses the development of arthritis [13]. These results support the idea that different *Prevotella* species have different effects on arthritis.

## 2. Microbiota and RA

**Figure 1:**
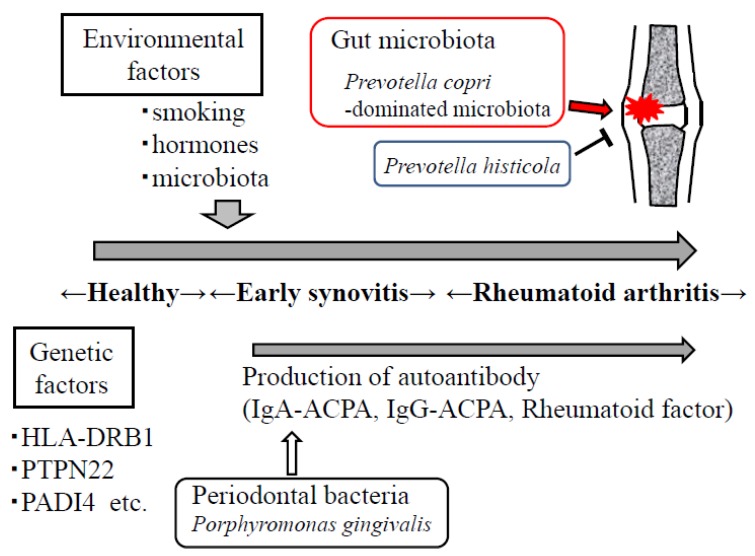
Environmental factors such as smoking, hormones, gut microbiota, and periodontal bacteria are important in the development of rheumatoid arthritis. *Prevotella copri*-dominated microbiota may contribute to the development of arthritis, whereas *P. histicola* suppresses the onset of arthritis.

Rheumatoid arthritis is a chronic autoimmune inflammatory disease characterized by auto-antibody production and destruction of bone in multiple joints (Figure 1) [14]. Recent studies have demonstrated that over 100 genetic susceptibility loci are involved in RA [15,16]. However, the environmental factors that affect the development of RA are not fully understood. It was recently shown that an immunoglobulin A (IgA) anti-citrullinated protein antibody (ACPA) is detectable before the onset of arthritis [17,18], suggesting that RA originates at mucosal sites, such as the oral cavity and the gut. *Porphyromonas gingivalis*, a major pathogenic bacterium of periodontal diseases, may correlate with the development of RA [19,20], because this bacterium is the only known pathogen that expresses a peptidylarginine deiminase and may be related to ACPA [21].

The gut microbiota contains the largest abundance of microorganisms in our body. The previous experiments in germ-free mice revealed that the gut microbiota shapes the intestinal immune system [22,23]. Recent studies in several countries have found that the composition of the intestinal microbiota is altered in patients with recent-onset RA. Commensal segmented filamentous bacteria (SFB) induce Th17 cells in the intestine and trigger arthritis in mice [24,25]. Therefore, the gut microbiota is thought to be an important environmental factor in the development of arthritis. 

## 3. Animal Models of Arthritis

Several animal studies have clearly demonstrated that gut microbiota plays an important role in arthritis development (Table 1). We and others have shown that SKG mice, which spontaneously develop arthritis under conventional conditions, did not develop arthritis in a germ-free (GF) environment [12,26]. We also showed that the mono-colonization of GF-SKG mice with *P. copri* was sufficient to induce arthritis with a fungal injection. 

Interleukin-1 (IL-1) receptor antagonist knock-out (IL1rn^−/−^) mice showed T cell -mediated arthritis in under a specific-pathogen-free (SPF) condition in response to excessive IL-1 signaling [27]. When the mice were reared under GF condition, they did not develop arthritis. However, the mono-colonization of these mice with *Lactobacillus* induced arthritis via activation of Toll-like receptor 2 (TLR2) and TLR4. 

K/BxN T cell receptor transgenic mice developed inflammatory arthritis, with increased numbers of Th17 cells in the small intestine and spleen [24]. The severity of arthritis and the titers of auto-antibodies directed against glucose-6-phospate isomerase were reduced when the mice were reared under GF condition. Mono-colonization with SFB was sufficient to cause the development of Th17 cell-dependent arthritis in this model. Therefore, a particular gut commensal microbiota is sufficient to induce arthritis in mice.

## 4. Human Microbiota in RA

The role of the gut microbiota in human RA is not fully understood. However, several studies have demonstrated that the composition of the intestinal microbiota is altered in RA patients (Table 2) [9,10,12].

Vaahtovuo et al. investigated the composition of the microbiota in patients with early RA or fibromyalgia using a technique based on flow cytometry, 16S rRNA hybridization, and DNA staining [9]. They found that the *Bacteroides fragilis* subgroup, the genus *Bifidobacterium*, and *Eubacterium rectale*–*Clostridium coccoides* are reduced in RA patients.

Using 16S rRNA gene sequencing, Scher et al. found that patients with recent-onset RA in North American populations carried an increased abundance of *P. copri* and a reduced abundance of *Bacteroides* in the gut [10]. We also confirmed that approximately one-third of Japanese patients with recent-onset RA had an increased abundance of *P. copri* in the gut [12]. 

Another study based on metagenomic shotgun sequencing showed that RA patients in China had an increased abundance of *L. salivarius* in the gut, on the tooth, and in the saliva [11]. However, the abundance of *P. copri* in the gut was only elevated in the first year after disease onset. The authors showed that the dysbiosis observed in RA patients partly improved after treatment with disease-modifying drugs.

## 5. Correlation between *Prevotella* and Arthritis

*Prevotella copri* was first isolated from human fecal samples in Japan [28]. It is an obligately anaerobic, non-spore-forming Gram-negative bacterium. Interestingly, Scher et al. showed that the abundance of *P. copri* was elevated in untreated recent-onset RA patients [10]. By contrast, the numbers of *P. copri* were reduced in patients with chronic RA, patients with psoriatic arthritis, and healthy volunteers. They also found that the relative abundance of *P. copri* in the intestine correlated with an absence of human leukocyte antigen (HLA)-DRB1. Moreover, *P. copri*–colonized mice displayed exacerbated colitis when they were treated with dextran sulfate sodium in their drinking water. However, the mechanistic link between the increased number of *P. copri* in the gut and arthritis is unknown. 

Therefore, we produced intestinal microbiota-humanized mice and analyzed the severity of their arthritis [12]. It has been reported that SKG mice develop Th17 cell-dependent autoimmune arthritis, clinically resembling human RA [29]. We used GF-SKG mice, which contain no bacterium in their gut. The GF-SKG mice showed no signs of arthritis when they were treated with a fungal component, suggesting that microbial stimuli are important for disease development. We colonized the GF-SKG mice with fecal samples from RA patients or healthy controls. The mice were kept in separate vinyl isolators. The SKG mice colonized with a *P. copri*–dominated microbiota from RA patients (RA-SKG mice) showed increased numbers of Th17 cells in the large intestine. We also confirmed that the abundance of *P. copri* increased in the large intestines of RA-SKG mice, but not in the small intestines. Intriguingly, the RA-SKG mice developed severe arthritis when they were injected with a low dose of zymosan (a fungal component). We also showed that lymphocytes from regional lymph nodes and large intestines of the RA-SKG mice produced high levels of IL-17 in response to the arthritis-related auto-antigen RPL23A. Moreover, bone marrow-derived dendritic cells stimulated with *P. copri* expressed high levels of IL-6 and IL-23 in an in vitro analysis. In addition, *P. copri*-monocolonized SKG mice showed Th17 cell-dependent arthritis development upon fungal injection, thus indicating that dysbiosis dominated by *P. copri* contributes to arthritis development. In the future, more intensive studies are needed to investigate whether *P. copri* elicits severe arthritis compared to other intestinal bacteria in in vivo experiments.

Very recently, Annalisa et al. used liquid chromatography–tandem mass spectrometry to identify an HLA-DR-presented peptide (T cell epitope) from a 27-kDa *P. copri* protein (Pc-p27) [30]. The peptide was detected in the synovial tissue, synovial fluid mononuclear cells, and peripheral blood mononuclear cells (PBMCs) of some RA patients. To investigate the Th1/Th17 responses to this peptide, the authors stimulated PBMCs from 40 RA patients with the peptide and evaluated the cytokines produced, with enzyme-linked immunosorbent assays (ELISA). They found that the levels of interferon γ (IFN-γ) were elevated in 42% of RA patients. However, PBMCs from only one RA patient showed increased IL-17 production. They also evaluated the IgG and IgA antibody responses to Pc-p27. IgG antibody responses were observed in 13% of new-onset RA patients and 20% of chronic RA patients. IgA antibody responses were also detected in approximately 10% of both new-onset and chronic RA patients. From these observations, they concluded that *P. copri* may contribute to the pathogenesis of RA. It will be interesting to analyze whether RA patients showing IgA response to Pc-p27 are also positive for IgA-ACPA.

Interestingly, several studies have shown that the genus *Prevotella* plays beneficial roles in our body. A recent study suggested that *P. histicola* isolated from the commensal bacteria in the human gut reduced the severity of collagen-induced arthritis in HLA-DQ8 mice [13]. *P. histicola* was isolated from the human duodenum and cultured on agar plates for further experiments. HLA-DQ8 mice were immunized with collagen and orally treated with *P. histicola.* The mice inoculated with *P. histicola* had a significantly reduced incidence of arthritis as a result of the suppression of the serum levels of several proinflammatory cytokines, such as IL-2, IL-17, and tumor necrosis factor α (TNF-α). The *P. histicola-*inoculated mice showed increased regulatory T cells in the gut and reduced antigen-specific Th17 responses. The researchers also demonstrated that sequences of *P. histicola* were very different from those of *P. copri*. These results suggest that not all *Prevotella* species are arthritogenic and that some of *Prevotella,* such as *P. histicola,* can suppress the development of arthritis.

## 6. Correlation between *Prevotella* and Insulin Resistance

A previous report also demonstrated a relationship between *P. copri* and glucose metabolism. One study showed that a barley-kernel-based bread (BKB) improved glucose metabolism by increasing the abundance of *P. copri* in the intestine [31]. When the researchers compared the composition of the microbiota in healthy individuals who responded or did not respond to the BKB intervention, they detected an increased abundance of *Prevotella* in the responders. Moreover, GF mice inoculated with the microbiota of the responders showed improved glucose metabolism, with an increased abundance of *P. copri* in the gut.

By contrast, another study in Denmark showed that *P. copri* induced insulin resistance [32]. The researchers investigated the correlation between the gut microbiota and serum metabolites in 277 non-diabetic individuals. They found that *P. copri* and *Bacteroides vulgatus* in the gut induced insulin resistance by increasing the serum levels of branched-chain amino acids (BCAA). To investigate whether *P. copri* directly induces insulin resistance, they inoculated C57BL/6J mice on a high-fat diet with *P. copri* and analyzed their glucose tolerance. The mice treated with *P. copri* showed increased levels of serum BCAA and insulin resistance. The researchers speculated that the different outcomes of the two studies were attributable to the different dietary regimens used.

## 7. Conclusions

We have reviewed the role of the gut microbiota in human RA and in mouse models of arthritis. Several studies have suggested that increased abundance of *P. copri* was observed in early RA patients. In contrast, another report showed other *Prevotella* species suppressed the induction of arthritis. Further studies are required to determine the precise mechanistic links between dysbiosis and the development of human RA. The modulation of the gut microbiota may offer a novel therapeutic or preventive approach to RA patients. 

## Figures and Tables

**Table 1:** Animal models of arthritis known to be correlated with intestinal bacteria.

Mice Strain	Environmental Condition	Mechanism of Involvement of Arthritis	Intestinal Bacteria Correlated with Induction of Arthritis	Ref.
K/BxN	GF:no arthritis SPF:arthritis	Production of GPI-antibody Th17 cell expansion in the intestine	SFB	[24]
IL-1ra^−/−^	GF:no arthritis conventional:arthritis	Activation of TLR2 and TLR4 Th17 cells↑ Treg cells↓	*Lactobacillus Bifidus*	[27]
SKG	GF, SPF:no arthritis conventional:arthritis	Production of auto-reactive T cells Activation of innate immunity by fungi	*Prevotella*-dominated microbiota	[12]

Note: GF: germ free, SPF: specific pathogen free, GPI: glucose-6-phospate isomerase, SFB: segmented filamentous bacteria, TLR: toll-like receptor, Treg cells: regulatory T cells.

**Table 2:** Altered composition of intestinal microbiota observed in human RA patients.

Country	Increased Bacteria	Reduced Bacteria	Method	Ref.
Finland	none	*Eubacterium rectale-Clostridium coccoides* group *Bacteroides fragilis* subgroup etc.	16S rRNA hybridization, DNA staining	[9]
United States	*Prevotella (Prevotella copri)*	*Bacteroides*	16S rRNA sequencing	[10]
China	*Clostridium asparagiforme Lactobacillus salivarius* etc.	*Veillonella, Haemophilus* etc.	Metagenomic shotgun sequence	[11]
Japan	*Prevotella (Prevotella copri) <1/3 of RA patients>*	*Bacteroides <1/3 of RA patients>*	16S rRNA sequencing	[12]
